# Slow-Growing Adrenal Incidentaloma Diagnosed as Pheochromocytoma

**DOI:** 10.1016/j.aed.2025.08.011

**Published:** 2025-08-25

**Authors:** Jeyavishnupriya Gopalakrishnan, Suprita Shrestha, Katrina Ann H. Corpuz, Egor Zakharchenko, Heng Yeh

**Affiliations:** Bassett Medical Center, Department of Medicine, Cooperstown, New York

## Visual Vignette

Case Presentation: A 73-year-old male with a history of chronic lymphocytic leukemia, atrial fibrillation, and hypertension presented with episodic palpitations and presyncope for the past 2 years. The spells were associated with headache, sweating, fine tremors, and increased blood pressure. He also had labile blood pressure readings on multiple occasions, ranging from the 70s/50s to the 250s/130s. He underwent multiple cardiac monitoring and an extensive cardiac workup without identifying the cause.

**Fig. 1:**
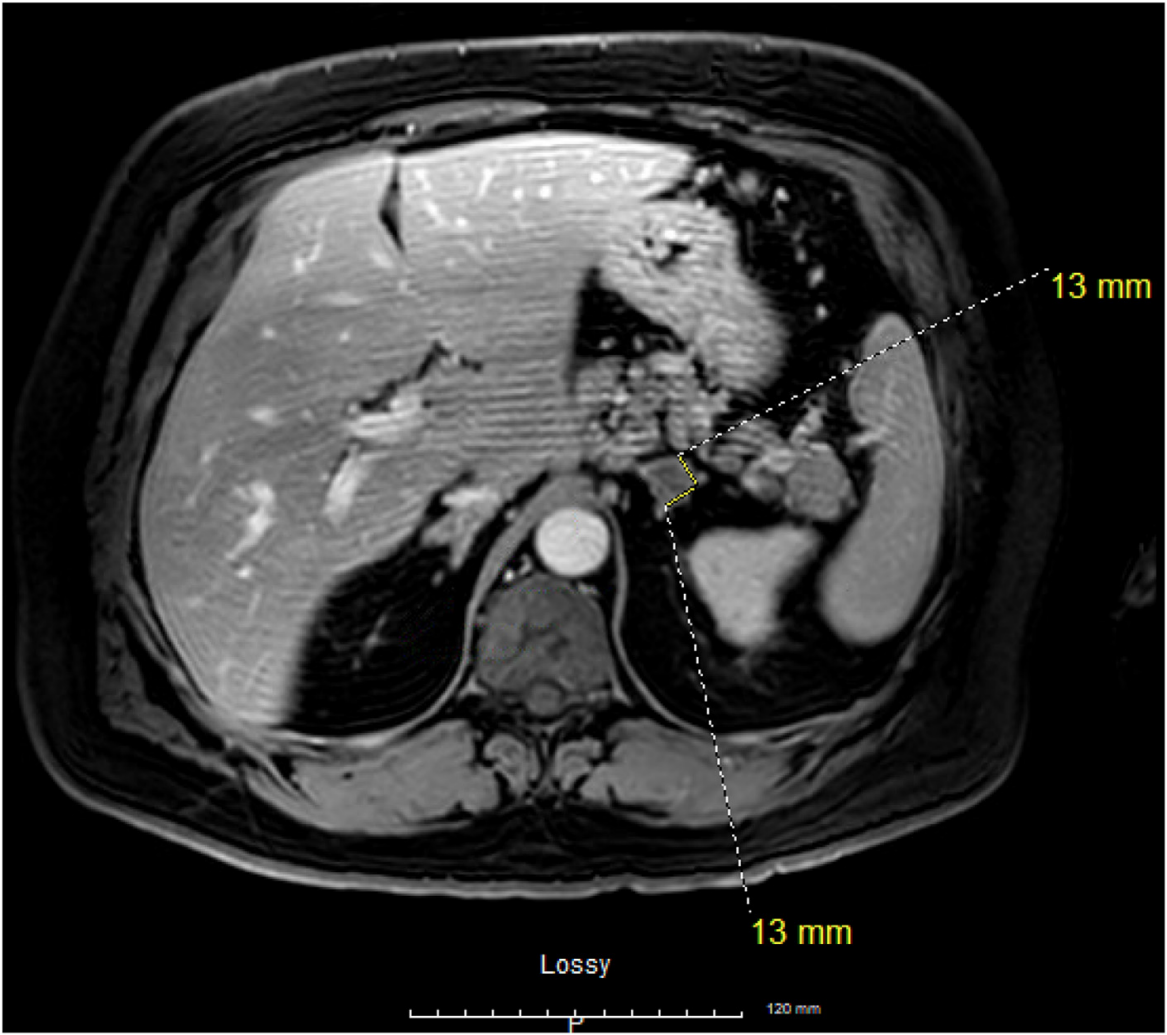
Magnetic resonance imaging (MRI) abdomen during cancer staging showing *left* adrenal tumor of size 13 × 13 mm.

**Fig. 2:**
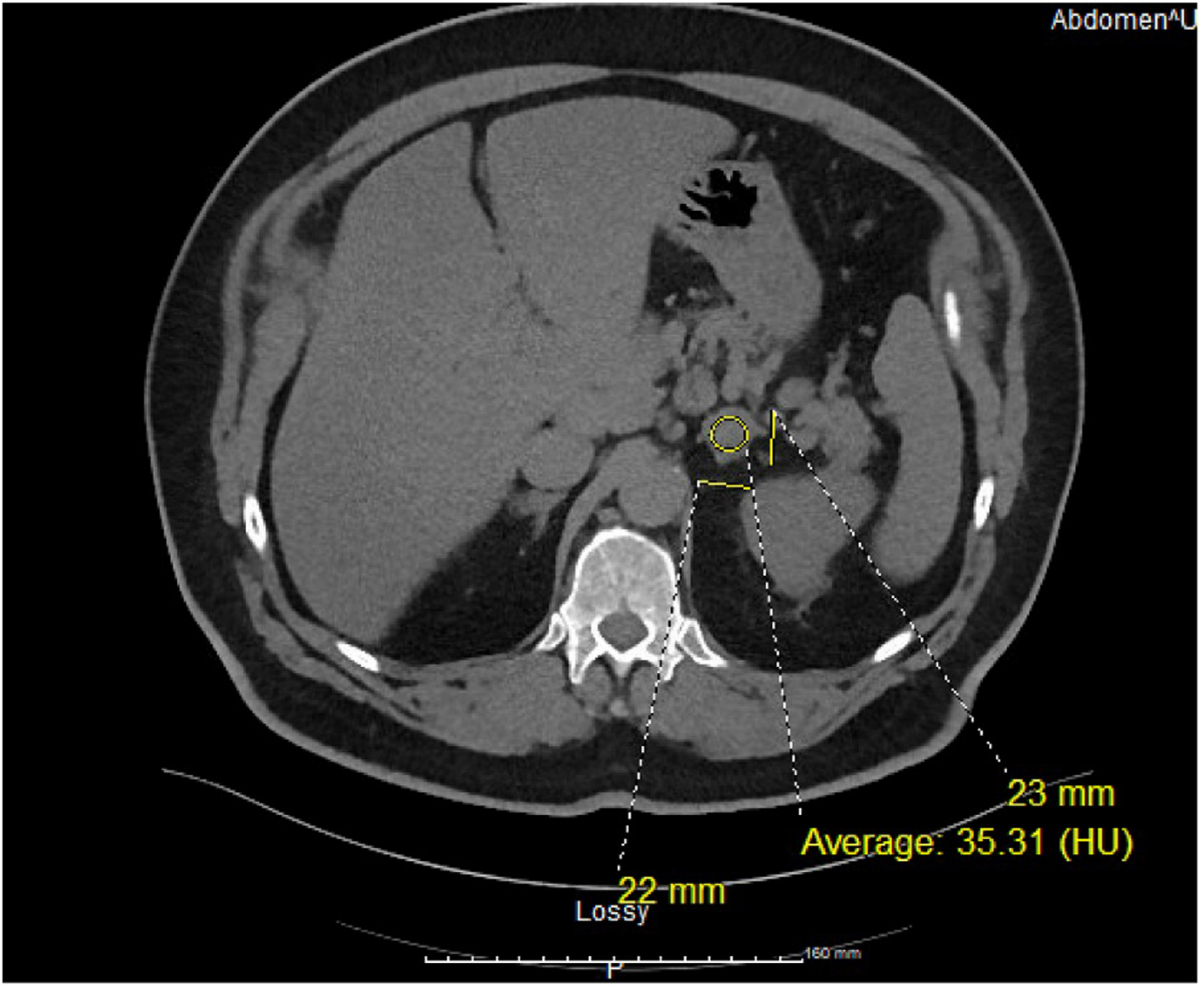
Noncontrast computed tomography (CT) abdomen and pelvis 3 years after initial cancer staging imaging showing *left* adrenal tumor sized 22 × 23 mm with 35.31 HU.

**Fig. 3:**
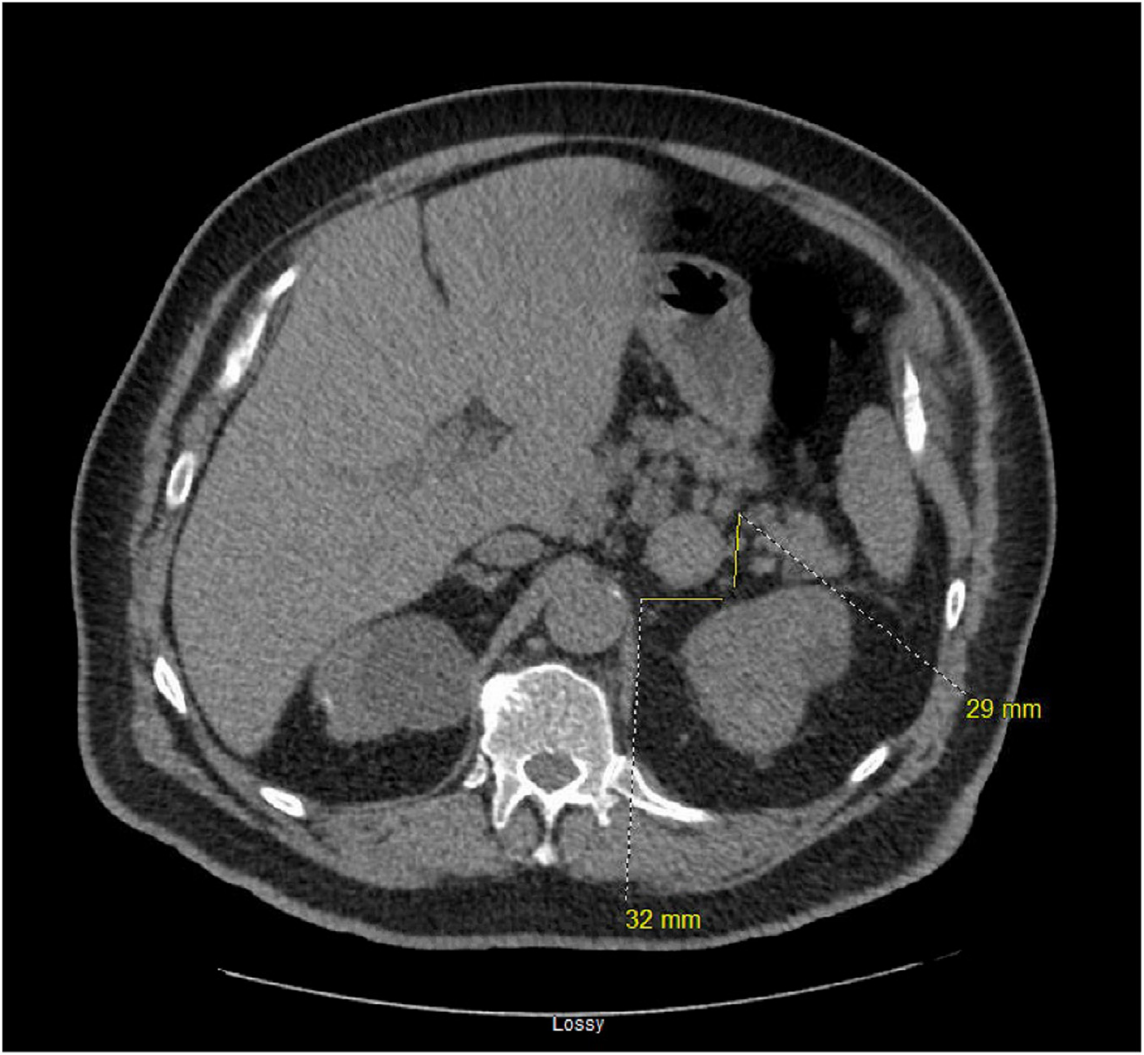
Noncontrast computed tomography (CT) abdomen and pelvis 5 years after initial cancer staging, showing progressive increase in size of the *left* adrenal tumor 32 × 29 mm.

On further chart review, a small left adrenal tumor was first documented 15 years ago without hormonal evaluation. Later, it was reported as a lipid-rich adenoma on an magnetic resonance imaging for his cancer staging imaging 5 years ago, sized 1.3 cm (Fig. 1). Subsequent cancer staging computed tomography (CT) documented it as a stable adrenal nodule, but it had been slowly growing by 0.3 cm every year and had progressed to a 3 cm tumor on the most recent CT (Figs. 2 and 3).

The hormonal tests revealed elevated plasma free metanephrine 2.5 nmol/L (<0.5 nmol/L), free normetanephrine 1.7 nmol/L (<0.9 nmol/L), and elevated 24-hour urinary normetanephrine 1239 mcg/24 h (<900 if hypertensive), total metanephrines 4207 mcg/24 h (<1300 if hypertensive), serum aldosterone level was <0.4 ng/dl (≤21 ng/dl).

What is the diagnosis?

Answer: Pheochromocytoma.

Pheochromocytomas represent 1.1% of adrenal tumors in the population, and the majority are discovered incidentally (61%). Pheochromocytomas are usually found as large tumors (median size 4 to 5 cm) with high Hounsfield units, >20 on unenhanced CT (97% to 99%), and slow growing around 3-5 mm yearly.^1^^,^^2^ In patients with incidental adrenal tumors, especially with a history of hypertension and HU >10 on unenhanced CT, biochemical assessment for primary hyperaldosteronism and pheochromocytoma is suggested.^1^ Follow-up imaging in 3 to 12 months in lipid-poor adenomas, and repeat biochemical testing for pheochromocytoma should be considered in case of tumor growth.^1^ Our case had imaging characteristics on CT for pheochromocytoma but didn’t undergo biochemical testing upon initial evaluation.

Perioperative medical management before surgery with alpha-adrenergic blockers is recommended to prevent perioperative cardiovascular complications, and dose titration should be based on goal blood pressure to <110-120/80. Ideally, it should be started 7-14 days before the surgery.^2^^,^^3^

He was started on doxazosin and underwent laparoscopic left adrenalectomy 1 month afterward. Pathology report of the excised tumor is consistent with pheochromocytoma. His symptoms resolved completely with normalization of plasma catecholamines after surgery.

## Patient Consent

Informed consent was obtained from the patient for the publication of this case and associated images.

## Disclosure

The authors have no conflicts of interest to disclose.
